# Evaluating the attrition, fabric integrity and insecticidal durability of two dual active ingredient nets (Interceptor^®^ G2 and Royal^®^ Guard): methodology for a prospective study embedded in a cluster randomized controlled trial in Benin

**DOI:** 10.1186/s12936-023-04708-x

**Published:** 2023-09-16

**Authors:** Corine Ngufor, Augustin Fongnikin, Josias Fagbohoun, Abel Agbevo, Thomas Syme, Juniace Ahoga, Manfred Accrombessi, Natacha Protopopoff, Jackie Cook, Thomas S. Churcher, Germain Gil Padonou, Renaud Govoetchan, Martin Akogbeto

**Affiliations:** 1 London School of Hygiene and Tropical Medicine (LSHTM), London, WC1E 7HT UK; 2 Centre de Recherches Entomologiques de Cotonou (CREC), Cotonou, Benin; 3 Panafrican Malaria Vector Research Consortium (PAMVERC), Cotonou, Benin; 4 MRC Centre for Global Infectious Disease Analysis, Infectious Disease Epidemiology, Imperial College London, Norfolk Place, London, W2 1PG UK

**Keywords:** Durability, Attrition, Fabric integrity, Bioefficacy, Experimental hut trials, Insecticide Treated Net, Pyriproxyfen, Chlorfenapyr, Alpha-cypermethrin, Dual active ingredient nets, Interceptor^®^, Interceptor^®^ G2, Royal Guard^®^

## Abstract

**Background:**

Following the World Health Organization (WHO) endorsement of dual active ingredient (AI) nets, an increased uptake of pyrethroid-chlorfenapyr and pyrethroid-pyriproxyfen nets is expected. Studies evaluating their physical and insecticidal durability are essential for making programmatic and procurement decisions. This paper describes the methodology for a prospective study to evaluate the attrition, fabric integrity, insecticidal durability of Interceptor^®^ G2 (alpha-cypermethrin-chlorfenapyr) and Royal Guard^®^ (alpha-cypermethrin-pyriproxyfen), compared to Interceptor^®^ (alpha-cypermethrin), embedded in a 3-arm cluster randomized controlled trial (cRCT) in the Zou Department of Benin.

**Methods:**

Ten clusters randomly selected from each arm of the cRCT will be used for the study. A total of 750 ITNs per type will be followed in 5 study clusters per arm to assess ITN attrition and fabric integrity at 6-, 12-, 24- and 36-months post distribution, using standard WHO procedures. A second cohort of 1800 nets per type will be withdrawn every 6 months from all 10 clusters per arm and assessed for chemical content and biological activity in laboratory bioassays at each time point. Alpha-cypermethrin bioefficacy in Interceptor^®^ and Royal Guard^®^ will be monitored in WHO cone bioassays and tunnel tests using the susceptible *Anopheles gambiae* Kisumu strain. The bioefficacy of the non-pyrethroid insecticides (chlorfenapyr in Interceptor^®^ G2 and pyriproxyfen in Royal Guard^®^) will be monitored using the pyrethroid-resistant *Anopheles coluzzii* Akron strain. Chlorfenapyr activity will be assessed in tunnel tests while pyriproxyfen activity will be assessed in cone bioassays in terms of the reduction in fertility of blood-fed survivors observed by dissecting mosquito ovaries. Nets withdrawn at 12, 24 and 36 months will be tested in experimental hut trials within the cRCT study area against wild free-flying pyrethroid resistant *An. gambiae *sensu lato to investigate their superiority to Interceptor^®^ and to compare them to ITNs washed 20 times for experimental hut evaluation studies. Mechanistic models will also be used to investigate whether entomological outcomes with each dual ITN type in experimental hut trials can predict their epidemiological performance in the cRCT.

**Conclusion:**

This study will provide information on the durability of two dual AI nets (Interceptor^®^ G2 and Royal Guard^®^) in Benin and will help identify suitable methods for monitoring the durability of their insecticidal activity under operational conditions. The modelling component will determine the capacity of experimental hut trials to predict the epidemiological performance of dual AI nets across their lifespan.

## Background

Insecticide-treated nets (ITNs) are the main malaria control and prevention intervention with over two and a half billion nets distributed globally between 2004 and 2021 [1]. Pyrethroids were for almost 3 decades the only insecticide class used on ITNs due to their safety profile, low cost and rapid activity against susceptible malaria mosquitoes. Modelling studies indicated that the reductions in malaria burden seen between the years 2000–2015 were largely due to large scale deployment of these nets especially in moderate to high transmission settings [2]. However, progress against malaria has stalled in recent years which is mostly attributed to stalling funding and the emergence, rapid spread and increasing intensity of resistance to pyrethroids in malaria vector mosquitoes across Africa [1, 3, 4], that undermines the impact of pyrethroid-only ITNs.

In response to the threat of pyrethroid-resistance, dual active ingredient (AI) ITNs treated with a mixture of a pyrethroid and a non-pyrethroid insecticide or compound to which local vector populations are largely susceptible have been developed for malaria vector control. Three new ITN types, pyrethroid-piperonyl butoxide (PBO), pyrethroid-chlorfenapyr and pyrethroid-pyriproxyfen ITNs, were recently added to the World Health Organization (WHO) list of prequalified vector control products after demonstrating improved impact against pyrethroid-resistant malaria vectors compared to pyrethroid-only nets [5]. Pyrethroid-PBO ITNs were the first new generation ITN class to further demonstrate improved public health value for control of clinical malaria compared to pyrethroid-only nets in cluster randomized controlled trials (cRCT) [6, 7] and based on these findings, they were conditionally recommended by the WHO for deployment in lieu of pyrethroid-only nets in pyrethroid-resistant areas in 2017 [8, 9]. Pyrethroid-chlorfenapyr and pyrethroid-pyriproxyfen ITNs are currently being evaluated in cRCTs in Benin and Tanzania for their improved public health value compared to pyrethroid-only nets over 3 years of operational use [10]. Based on results up to 24 months post-ITN distribution from these trials [11, 12], the WHO has recently released a strong recommendation for pyrethroid-chlorfenapyr ITNs over pyrethroid-only nets and a conditional recommendation for pyrethroid-pyriproxyfen nets over pyrethroid-only ITNs for malaria control in areas where vectors have become resistant to pyrethroids [9].

Following the WHO recommendation, an increased uptake of pyrethroid-chlorfenapyr and pyrethroid-pyriproxyfen nets is expected in the coming years. However, to be considered long-lasting, new ITNs need to be evaluated for their physical and insecticidal durability over 3 years of operational use [13–15]. Studies have shown that the durability of ITNs may vary widely from one community to another and between different product brands [16, 17]. ITN durability studies are useful for planning the replacement of worn-out nets and making programmatic and procurement decisions [14]. Modelling studies have suggested that ITNs are discarded more quickly than the 3 years presumed by country policies, and this is driven by several factors including the physical durability of the ITN brand [18]. Current WHO guidelines for assessing the attrition, fabric integrity, chemical content and insecticidal durability of ITNs under operational use [13, 15] were developed when pyrethroids were the only insecticides applied on ITNs and will need to be updated to cover new dual AI ITNs. This will require the development of standardized methods for assessing the insecticidal durability of non-pyrethroid insecticides like chlorfenapyr and pyriproxyfen on nets. Unlike pyrethroids that knockdown and kill mosquitoes, chlorfenapyr is a pyrrole insecticide that acts on the insect mitochondria to slowly induce death [19], while pyriproxyfen is an insect growth regulator that sterilizes adult female mosquitoes leading to a substantial reduction in offspring [20, 21]. Laboratory bioassay methods and strains used for assessing their bioefficacy on ITNs must, therefore, be aligned with the mode of action of these insecticides. In addition to bioassays (cone bioassays and tunnel tests), experimental hut trials have also been proposed as a suitable method for investigating the insecticidal durability of ITNs under operational conditions [22, 23]. Using hut trials, ITNs can be assessed against wild free-flying mosquito vectors providing more realistic estimates of their capacity to provide personal and community protection to householders as they age.

Experimental hut trials are typically used for assessing the entomological efficacy and wash resistance of ITNs under semi-field conditions for the WHO prequalification and for determining the non-inferiority of new ITN products to a similar product that has demonstrated empirical evidence of improved epidemiological impact in cRCT [13, 24]. For such studies, nets are artificially aged by washing up to 20 times to mimic naturally aged nets at the end of their operational life (3 years) and their entomological performance in experimental huts is used as a proxy for their disease control capacity. Cluster randomized controlled trials are the gold standard evidence for disease control interventions, however, because they are expensive and time consuming, hut trials have been used as a potentially more cost-effective high throughput alternative to support policy and decision-making to guide vector control product uptake [24, 25]. Given the increasing importance of experimental hut trials in the vector control product development process, studies establishing their capacity to predict the epidemiological performance of vector control products in cRCT are essential. This will ideally require the generation of experimental hut data alongside cRCT, which unfortunately has not been done in the past. Recent studies have indicated the capacity of mechanistic malaria transmission dynamics mathematical models to reliably predict the epidemiological performance of current widely used pyrethroid-only nets using data derived from experimental hut trials [25]. As the demand for dual AI ITNs increases, it is essential to establish the capacity of experimental hut data to predict their epidemiological performance to help speed up product development and decision-making for their deployment. The on-going cRCT in Benin provides a unique opportunity given that hut trials can be performed in parallel within the study area of the cRCT trial, facilitating modelling studies to investigate correlations between entomological data and epidemiological performance with clinical malaria endpoints.

This paper describes the methodology for a prospective longitudinal study investigating the attrition, physical and insecticidal durability of two dual AI ITNs (Interceptor® G2 and Royal Guard^®^) compared to a pyrethroid-only ITN (Interceptor^®^), embedded in a cRCT in Benin. We also discuss key issues around the evaluation of the insecticidal durability of dual AI ITNs. Prospective follow up surveys will be used to assess ITN attrition and fabric integrity and modified laboratory bioassays and experimental hut trials to evaluate the durability of the insecticidal efficacy of the non-pyrethroid component of both types of dual AI ITNs over 3 years of household use during the cRCT. Experimental hut trials will also be performed to investigate the capacity of ITN washing following standard WHO ITN evaluation procedures to predict the performance of naturally aged field nets of each ITN type. Finally, mechanistic models will be used to investigate whether entomological outcomes with each dual ITN type from hut trials can be used to predict their epidemiological performance in the cRCT.

## Overall aim of the study

To assess the physical and insecticidal durability of two dual AI nets; Interceptor^®^ G2 (alpha-cypermerthrin plus chlorfenpayr) and Royal Guard^®^ (alpha-cypermethrin plus pyriproxyfen) compared to a standard alpha-cypermethrin only net (Interceptor^®^) over 3 years of community use during a randomized-controlled trial in the Zou Region of Benin.

## Study objectives

- To determine the survivorship, attrition and fabric integrity of Interceptor^®^ G2 and Royal Guard^®^ compared to a standard alpha-cypermethrin-only net (Interceptor^®^) over 3 years of household use in communities in Benin
- To investigate insecticidal activity and chemical content of Interceptor^®^ G2 and Royal Guard^®^ compared to Interceptor^®^ over 3 years of household use in communities in Benin.
- To investigate householders’ preferences and perceptions of the different ITN fabric types (polyethylene for Royal Guard^®^ and polyester for Interceptor^®^) and it’s impact on ITN retention over 3 years of use in communities in Benin
- To compare the performance of nets washed 20 times for experimental hut trial evaluations to 3 year old field aged nets.
- To investigate correlations between the entomological performance in hut trials and the epidemiological performance of each ITN type in the cRCT in Benin using mathematical models.

## Methods

### Study arms

The study consists of three arms evaluating the durability of two dual AI ITNs (Interceptor^®^ G2 and Royal Guard^®^) compared to a standard pyrethroid-only ITN Interceptor^®^. Study nets are all blue, rectangular, and sizes of 1.8 m long, 1.8 m wide, and 1.8 m high (Interceptor^®^ and Interceptor^®^ G2) and 1.8 m long, 1.8 m wide, and 1.6 m high (Royal Guard^®^). A description of the specifications of the three ITN types is provided below:

1. Interceptor^®^ G2 (BASF AGRO B.V.), is a WHO-prequalified 100-denier, polyester ITN coated with a mixture of alpha-cypermethrin and chlorfenapyr at target concentrations of 100 mg/m^2^ (± 25%) and 200 mg/m^2^ (± 25%), respectively. The bursting strength of the fabric is ≥ 405 kPa while the mass per unit area is 40 g/m^2^ (± 10%) for 100 denier yarn.
2. Royal Guard^®^ (Disease Control Technologies, LLC, USA), is a WHO-prequalified 100-denier, polyethylene ITN incorporated with a mixture of alpha-cypermethrin and pyriproxyfen at target concentrations of 261 mg/m^2^ (± 25%) and 225 mg/m^2^ (± 25%) respectively. The bursting strength of the fabric is ≥ 405 kPa while the mass per unit area is 45 g/m^2^ (± 10%) for 150 denier yarn.
3. Interceptor^®^ (BASF AGRO B.V.), is a WHO-prequalified 100-denier, polyester ITN coated with alpha-cypermethrin at target concentrations of 200 mg/m^2^ (± 25%). The bursting strength of the fabric is ≥ 405 kPa while the mass per unit area is 40 g/m^2^ (± 10%) for 100 denier yarn. Interceptor® is the control arm of the trial against which Interceptor^®^ G2 and Royal Guard® are compared.

### Study setting

This ITN durability study is embedded in a cRCT performed in the Cove, Zagnanado, and Ouinhi districts (COZO), of the Zou department of central Benin (7°11′N 1°59′E). The design of the cRCT has been published previously [10]. The study area consists of 123 villages with approximately 54,000 households and a population size of 220,000 inhabitants. Baseline studies performed in 2019 as part of the cRCT demonstrated a malaria prevalence of 43.5% [26] and high intensity of resistance to pyrethroids but no resistance to chlorfenapyr and pyriproxyfen in main malaria vectors in the study area; *Anopheles gambiae* and *Anopheles coluzzii* [27].

### Study design

**Fig. 1:**
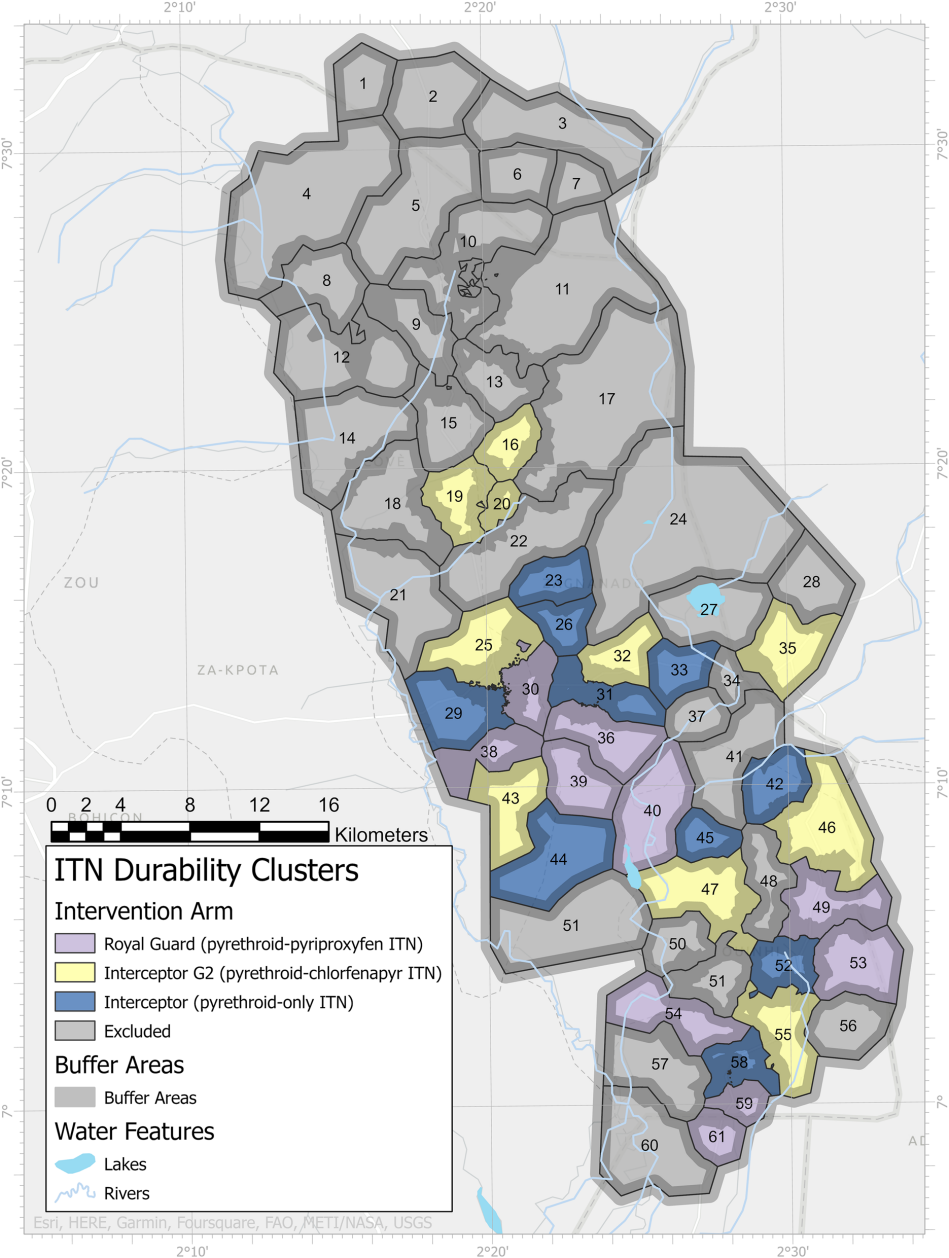
Map of study area of cluster randomized controlled trial showing ITN durability assessment clusters. Ten clusters were randomly selected from each study arm

**Fig. 2:**
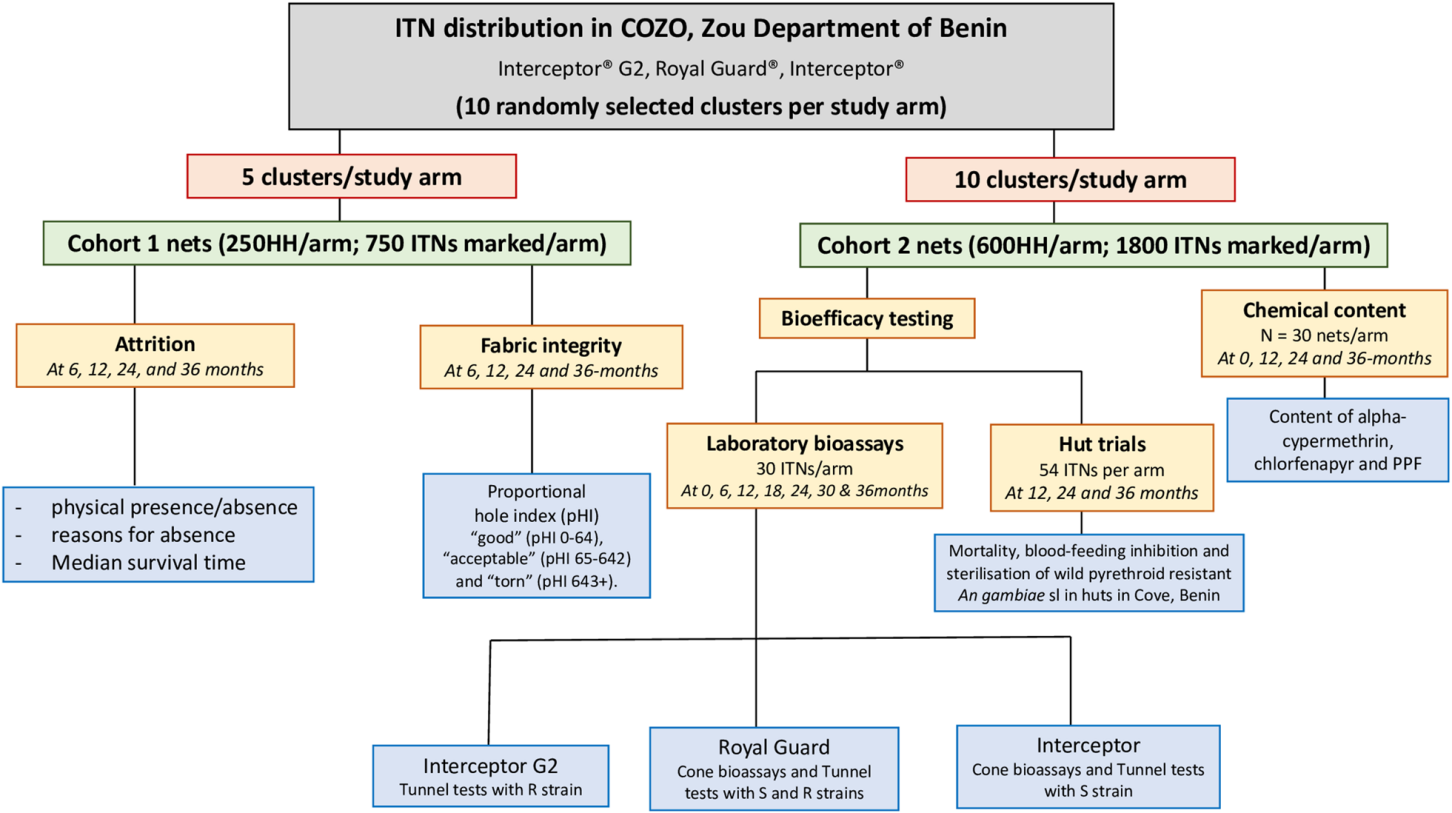
Flow chart of trial activities and outputs for monitoring the physical and insecticidal durability of Interceptor G2, Royal Guard and Interceptor in Benin. Two cohorts of nets will be followed per study arm; cohort 1 nets will be assessed for ITN attrition and fabric integrity while cohort 2 nets will be withdrawn (and replaced) and assessed for bioefficacy and chemical content

The study area was divided into 60 clusters that were randomly assigned to the three arms of the cRCT using restricted randomization. Study nets are identical in appearance and participating households are blinded to the type of ITN they received. Field data collectors are also blinded to the allocation of households to the study arms. Ten clusters from each study arm have been randomly selected for the durability assessment (Fig. 1). Following ITN distribution, households in these clusters will be visited to mark and prospectively follow 2 separate cohorts of ITNs per arm (Cohorts 1 and 2) every 6–12 months to monitor their physical and insecticidal durability over a period of 36 months. Cohort 1 will consist of at least 750 nets from ~ 250 randomly selected households per arm (assuming 3 ITNs are distributed per household) from 5 study clusters per study arm. These nets will be followed for ITN survivorship and fabric integrity at 6-, 12-, 24- and 36-months post ITN distribution. Cohort 2 will consist of ~ 1800 nets per study arm from ~ 600 households from all 10 randomly selected clusters (60 households per cluster) per study arm to be withdrawn (and replaced with new nets) for assessment of insecticidal activity in laboratory bioassays and experimental hut trials, content of each active ingredient and for other studies. Cohort 1 and cohort 2 nets will receive separate identification numbers and would be sampled from different households to prevent the risk of destructive sampling of nets intended for ITN survivorship studies (cohort 1). Figure 2 provides a summary of the study design and studies to be performed at each timepoint.

### Household sensitization and marking of study nets

Households and nets for both cohorts of the durability study will be drawn from the selected clusters of each arm of the cRCT using the ITN distribution list of the trial. Within the first month after ITN distribution, field workers will visit the selected households for each cohort of ITNs to recruit and mark study nets available in them. The number of households enrolled will be increased until the ITN sample size for each cohort is achieved (750 for cohort 1 and 1800 for cohort 2). Using a wash resistant marker, a unique code will be written on the study net to indicate the study arm, cluster ID, household ID and the cohort to which it belongs. During this visit, the durability study will be explained to the householders in their local language and the study team will assist them in hanging up their nets if needed. Only nets which have been successfully hung up and are in use in selected households at the start of the trial will be included in the durability study. GPS coordinates and characteristics of each household (housing type and socio-economic characteristics) included in the durability assessment will also be recorded.

### Follow up surveys for cohort 1 nets

#### Follow-up for ITN attrition

All households in cohort 1 (at least 750 nets/arm) will be visited at 6-, 12-, 24- and 36-months post ITN distribution and nets assessed for attrition at each timepoint by recording the physical presence/absence of each net that was marked in the household. Where a net is not found, the householder will be asked about the reason for the loss of the net, e.g. given away, sold, stolen, worn out and disposed of. Any net which has never been used, will be recorded, and excluded from the analysis. During these surveys, a questionnaire adapted from WHO guidelines will be used to collect data on the number of washes and washing practices for the nets found in each household.

ITN retention may also be affected by householders’ preference of the fabric type used in the formulation of the net. A low preference for polyethylene nets has been previously observed in Benin by the National Malaria Control Programme (NMCP), which may differentially affect the retention of Royal Guard^®^ (polyethylene) compared to Interceptor^®^ and Interceptor^®^ G2 (polyester). As part of these follow up surveys, a short questionnaire will be administered to assess householders’ perception of the ITN fabric types and changes in size of each ITN type to determine whether this may have impacted their decision to discard the net. A subset of ITNs of each type at 0, 12, 24 and 36-months post-distribution (minimum 100 per ITN type) will also be measured to directly examine changes in ITN size over time.

#### Follow-up for ITN fabric integrity

Fabric integrity (hole index) and condition will be observed on all nets of cohort 1 at 6-, 12-, 24- and 36-months post distribution. Where the minimum number (250) of nets for fabric integrity is not attainable from the longitudinal cohort (cohort 1) due to loss, ITNs sampled destructively from the same clusters for bio-efficacy would first be examined for fabric integrity prior to the bio-efficacy studies. The survey team will inspect the nets outside in broad day light to determine the hole index using a portable frame over which the net can be draped during inspection. The nets will be returned to the family right after the inspection. Hole number, position and size will be assessed on each ITN panel (roof and sides) using hole assessment sheets and classified into 4 sizes (size 1: 0.5-2 cm, Size 2: 2–10 cm, Size 3: 10-25 cm, and size 4: > 25 cm). Physical integrity will be measured as the proportionate hole index (pHI), categorized based on recommended cut-off points into “good” condition (pHI 0–64), “acceptable” condition (pHI 65–642) and “torn” (pHI 643 +) defined in WHO guidelines [13].

### Bioefficacy studies and chemical analysis with cohort 2 nets

Nets belonging to cohort 2 will be randomly selected per arm for bioefficacy studies and chemical analysis. Nets of each type (Interceptor^®^ G2, Royal Guard^®^ and Interceptor^®^) will be destructively sampled from households at 6, 12, 18-, 24-, 30- and 36-months post-distribution and replaced with new nets of the same type at each sampling point. They will be subjected to laboratory bioassays (WHO cone bioassays and tunnel tests where necessary) and experimental hut trials to monitor entomological efficacy against mosquito vectors and to chemical analysis to monitor changes alphacypermethrin, chlorfenapyr and pyriproxyfen content. For laboratory bioassays, net pieces measuring 30cmx30cm will be cut from each net following WHO guidelines [13]. Nets withdrawn at annual time points (12, 24 and 36 months) will be tested in experimental huts before laboratory bioassays. The number of whole ITNs of each type to be subjected to each type of study at each timepoint is summarized in Table 1 below.

**Table 1:** Number of Interceptor^®^ G2, Royal Guard^®^ and Interceptor^®^ ITNs to be sampled from Cohort 2 at each timepoint

Months post ITN distribution	No of ITNs to withdrawn for bioefficacy studies	No. of ITN to test in hut trials	No. of ITNs to test in lab bioassays	No. of pieces per ITN	Total number of pieces for bioassays	No. of pieces for chemical analysis^b^	ITNs sampled for other studies^c^	
0	30	–	30	5	150	150		
6	30	–	30	4	120	–		
12	54^a^	54^a^	30	4	120	120	80	
18	30	-	30	4	120	-		
24	54^a^	54^a^	30	4	120	120	80	
30	30	–	30	4	120	–		
36	54^a^	54^a^	50	4	200	200	80	
Total	282				950	590	240	

^a^These nets will be tested in hut trials prior to laboratory bioassays and chemical analysis. Hut trials will only be performed with nets withdrawn at 12, 24 and 36 months

^b^Net pieces preserved for chemical analysis will be obtained from adjacent positions on the same ITNs cut for bioassays

^c^These nets will be samples from cohort 2 nets but will be used in other studies

#### Laboratory bioassays and strain selection

Cone bioassays and tunnel tests will be performed at the CREC-LSHTM GLP-certified facility to monitor the bioefficacy of each active ingredient in each ITN brand using ITN pieces obtained from field collected nets at each time point. Current WHO ITN durability guidelines were tailored towards pyrethroid-only nets and therefore do not describe methods for assessing the bioefficacy of non-pyrethroid chemicals on ITNs [13]. Unlike pyrethroids that require a pyrethroid-susceptible *Anopheles* strain for durability bioassays, evaluation of the insecticidal activity of chlorfenapyr in Interceptor^®^ G2 and pyriproxyfen in Royal Guard® will require the use of a pyrethroid-resistant strains that is susceptible to these insecticides to reduce any confounding effects from the pyrethroid component.

**Fig. 3:**
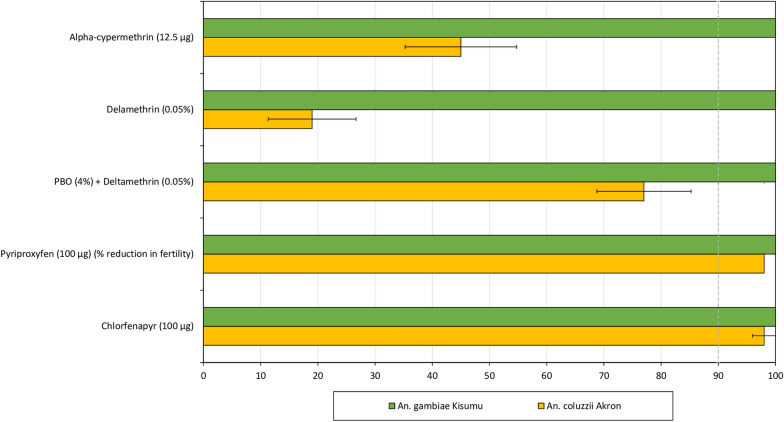
Susceptibility of the pyrethroid-resistant *Anopheles coluzzii* Akron strain to insecticides in study nets. Chlorfenapyr, pyriproxyfen and alpha-cypermethrin exposures were performed in WHO bottle bioassays while deltamethrin exposures were performed in WHO filter paper tube tests. Approximately 100 mosquitoes of each strain were exposed for 1 h to each insecticide in replicates of 25. For pyriproxyfen, mosquitoes were blood-fed before exposure and their ovaries dissected after 72 h to assess ovary development

Following a series of preliminary laboratory bioassays, the pyrethroid resistant *An. coluzzii* Akron strain (BEI resources) maintained at the CREC-LSHTM insectary since 2018 was identified as a suitable strain for monitoring the bioefficacy of chlorfenapyr and pyriproxyfen in Interceptor^®^ G2 and Royal Guard^®^ ITNs from the trial. Preliminary WHO susceptibility bottle bioassays [28, 29] performed with alpha-cypermethrin (12.5μg), chlorfenapyr (100μg) and pyriproxyfen (100μg) showed that the strain was resistant to pyrethroids but susceptible to chlorfenapyr and pyriproxyfen (Fig. 3). Mortality with deltamethrin in WHO tube assays increased from 19 to 77% demonstrating the partial involvement of mixed function oxidases in pyrethroid resistance in the Akron strain. Dissection of ovaries of blood-fed *An. coluzzii* Akron mosquitoes exposed to pyriproxyfen treated bottles revealed a 98% reduction in mosquito fertility relative to unexposed mosquitoes thus showing susceptibility to pyriproxyfen. The WHO cone bioassays performed with Akron mosquitoes using unwashed and washed pieces of the pyrethroid-only ITN (Interceptor^®^) showed high survival (> 60%) demonstrating that the impact of chlorfenapyr and pyriproxyfen in Interceptor^®^ G2 and Royal Guard^®^ will be less confounded by the alphacypermethrin component in these nets when tested in laboratory bioassays using this strain.

Based on results from these preliminary bioassays, the testing plan in Table 2 will be used in laboratory bioassays for monitoring the bioefficacy of each active ingredient in the different ITN brands in the trial. In line with WHO guidelines, ITN bioefficacy at each timepoint will be expressed in terms of the proportion of ITNs passing the efficacy criteria for each active ingredient in cone bioassays or tunnel tests.

**Table 2:** Summary of laboratory bioassay methodology and outcome measures for assessing the insecticidal durability of Interceptor^®^ G2 and Royal Guard^®^ in Benin compared to Interceptor^®^

ITN type	Brand name	Active ingredient	Mosquito strain	Mosquito age and status (N)	Primary test method (exposure time)	Key outcome measures	ITN efficacy criteria	Remarks/additional tests
Pyrethroid-only	Interceptor	alpha-cypermethrin	Susceptible *An. gambiae* s.s. Kisumu	unfed, 3–5 days old (40–50/net)	cone bioassays (3 min)	Knockdown, 24 h Mortality	knock-down ≥ 95% or mortality ≥ 80%	Tunnels for failed nets
Pyrethroid-chlorfenapyr	Interceptor G2^a^	alpha-cypermethrin	not tested	not tested	not tested	not tested	not tested	not tested due to lower dose of pyrethroid
		chlorfenapyr	Pyrethroid-resistant *An. coluzzii* Akron	unfed, 5–8 days old (100/net)	Tunnel tests (overnight)	72 h Mortality and Blood-feeding inhibition	Mortality ≥ 80% or Blood-feeding inhibition ≥ 80%	1 net piece per ITN
Pyrethroid- pyriproxyfen	Royal Guard	alpha-cypermethrin	Susceptible *An. gambiae* s.s. Kisumu	unfed, 5–8 days old (40–50/net)	cone bioassays (3 min)	Knockdown, Mortality	knock-down ≥ 95% or mortality ≥ 80%	Tunnels for failed nets
		pyriproxyfen	Pyrethroid-resistant *An. coluzzii* Akron	blood-fed, 5–8 days old (80–100/net)	cone bioassays (3 min) plus ovary dissection after 72 h	% reduction in fertility relative to control^b^	Reduction in fertility relative control	> 30% fertility required in control

^a^For Interceptor G2, given the low dose of alpha-cypermethrin, chlorfenapyr bioefficacy will be prioritised

^b^Mosquitoes presenting with Christopher stage V ovaries, will be considered fertile

Alpha-cypermethrin bioefficacy in Royal Guard^®^ and Interceptor^®^ will be assessed using the susceptible *An. gambiae* Kisumu strain in 3 min cone bioassays following current WHO protocols. At each timepoint, 40–50 unfed 3–5 days old *An. gambiae* Kisumu mosquitoes will be exposed to each whole Royal Guard^®^ and Interceptor^®^ ITN in replicates of 5 mosquitoes per cone and 2 cones per ITN piece. ITNs which fail to achieve WHO efficacy criteria in these cone bioassays (pooled knock-down ≥ 95% or pooled 24h mortality ≥ 80%) will be subjected to tunnel tests. For each ITN that failed in cone bioassays, only one ITN piece (with cone bioassay mortality that is closest to the mean) will be tested in tunnels. Approximately 100 unfed 5–8 days old susceptible *An gambiae* Kisumu will be exposed to each ITN piece in the tunnel tests and efficacy of alpha-cypermethrin in Interceptor^®^ and Royal Guard^®^ will be measured in terms of mortality after 24h (≥ 80%) or blood-feeding inhibition (≥ 90%).

Given the relatively low concentration of alpha-cypermethrin in Interceptor^®^ G2 (100 mg/m^2^), bioefficacy studies performed with the net will focus more on its chlorfenapyr component. The unsuitability of cone bioassays for assessing the efficacy of chlorfenapyr on ITNs has been demonstrated in several studies [19, 30] hence only tunnel tests will be used for evaluating the activity of chlorfenapyr in Interceptor^®^ G2 with the pyrethroid-resistant Akron strain. To improve the efficiency of the tunnel test bioassays, only 1 ITN piece per whole Interceptor^®^ G2 will be tested in tunnels. A total of ~ 100 unfed 5–8 days old pyrethroid resistant *An coluzzii* Akron will be exposed overnight in each tunnel test with Interceptor^®^ G2 and efficacy in tunnels will be measured in terms of mosquito mortality after 72h (≥ 80%) or blood-feeding inhibition (≥ 90%).

**Fig. 4:**
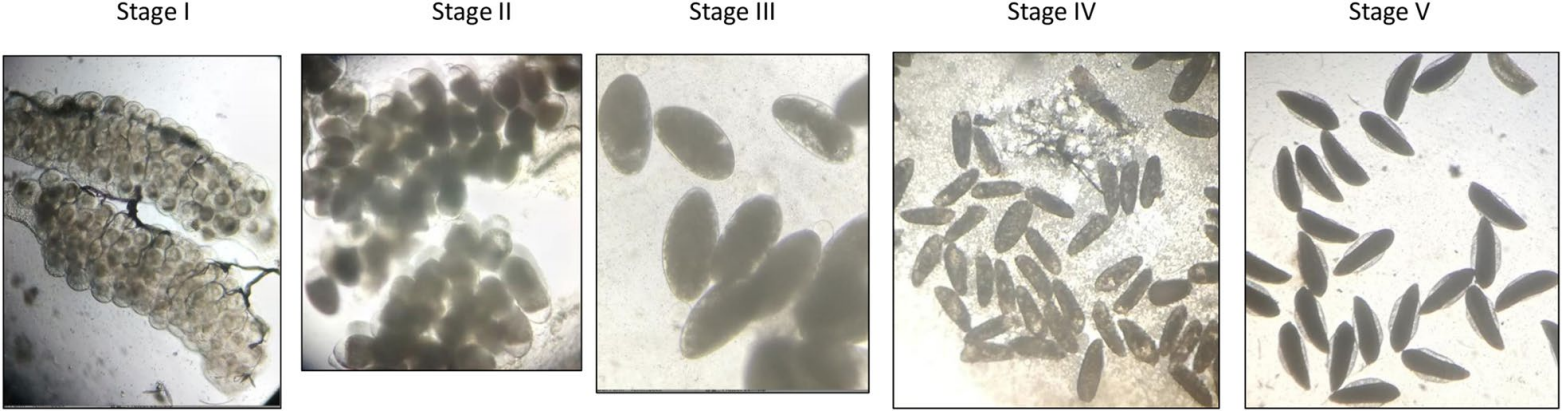
Developmental stages of Anopheline ovaries [31]. Mosquitoes with fully developed stage V ovaries (elongated boat-shaped eggs with lateral floats) are considered fertile while mosquitoes with stage I-IV ovaries containing underdeveloped eggs are considered infertile

For bioefficacy of pyriproxyfen in Royal Guard^®^, blood-fed 5–8 days old mosquitoes of the pyrethroid-resistant *An coluzzii* Akron strain will be exposed for 3 min in cone bioassays and assessed for the impact on ovary development using dissection. Dissections will be performed under microscope by trained technicians, 72h after exposure. Mosquitoes presenting with Christopher stage V ovaries will be classified as fertile (Fig. 4) [31] and the proportional reduction in fertility relative to control unexposed mosquitoes at each bioassay will be determined for each ITN. A total of 80–100 blood-fed mosquitoes will be tested against each Royal Guard^®^ ITN in replicates of 5 mosquitoes per cone and 4 cones per ITN piece. As there are currently no WHO defined cut-offs for the reproductive effect of PPF-treated ITNs in bioassays, the pass rate of Royal Guard® nets at each timepoint will be assessed based on their capacity to reduce the fertility of exposed mosquitoes relative to the untreated control. Only tests for which at least 30% of unexposed mosquitoes are found fertile will be considered valid. Efficacy in these cone bioassays will be considered the final endpoints for performance of pyriproxyfen in Royal Guard^®^; no further tests will be performed in tunnels with failed nets.

All bioassays will be performed at 27 ± 2 °C and 75% ± 10% relative humidity. For each ITN type, each active ingredient and each bioassay, a direct comparison will be made with a new unused net during bioassays. A new Interceptor^®^ net will be tested as positive control in bioassays evaluating pyriproxyfen in Royal Guard^®^ and chlorfenapyr in Interceptor^®^ G2. Prior to each bioassay round, susceptibility tests will be performed to monitor the suitability of the *An coluzzii* Akron strain. While efforts will be made to test nets withdrawn at all timepoints (every 6 months) in laboratory bioassays, given the volume of bioassays required, to improve efficiency, nets withdrawn at 0, 6, 12, 24 and 36 months post-ITN distribution will be prioritized over other timepoints.

### Chemical analysis

Net pieces preserved for chemical analysis at 0, 12, 24 and 36 months (590 pieces per net type) will be wrapped in Aluminium foil and stored at 4^°^c (± 2°c) and afterwards shipped to Centre Walloon de Recherches Agronomiques (CRA-W), Belgium, for detection of fabric weight and active ingredient content. The methods to be used for AI extraction have been described elsewhere [30]. Gas Chromatography with Flame Ionisation Detection (GC-FID) will be used to determine the content of each AI. ITN pieces from the same net will be pooled to provide a single chemical content reading per AI for each whole ITN per net type sampled at each time point.

### Experimental hut trials to assess insecticidal durability

To further assess the entomological superiority of Interceptor^®^ G2 and Royal Guard^®^ over Interceptor^®^, experimental hut trials will be performed with field collected ITNs at 12-, 24- and 36-months post ITN distribution at the Cove experimental hut station against wild free-flying pyrethroid-resistant *An. gambiae *sensu lato (*s.l*.). The Cove experimental hut station is situated in a large rice-growing field within the study area of the cRCT. The vector population at Cove exhibits intense resistance to pyrethroids [32], but is susceptible to chlorfenapyr and pyriproxyfen [30]. To provide some comparison to pyrethroid-PBO nets, PermaNet^®^ 3.0 nets (a WHO prequalified deltamethrin plus PBO ITN by Vestergaard Sarl) that were distributed by the NMCP in the Didja district (7^°^20’N 1^°^56’E) of the Zou Department, a neighbouring community to the cRCT study area, at the same time as the cRCT will also be marked at baseline and withdrawn at each annual timepoint and assessed in the experimental hut trials alongside Interceptor^®^ G2 and Royal Guard®. Three sets of hut trials will be performed: one with 12 months old field nets, the second with 24 months old field nets and the third with 36 months old field nets. Fifty-four (54) field nets of each brand will be tested in the huts at each annual timepoint for a total of 54 nights with each field net spending only 1 night in the experimental huts. Comparison will be made with new unused nets of each type. Six replicates of unused nets of each ITN type will also be tested, and each net will be given six 4 × 4cm holes prior to the trial, following WHO procedures. The hut trials will consist of the following 9 treatments:

1. Untreated net—6 replicates
2. Interceptor^®^ (new unused)—6 replicates
3. PermaNet^®^ 3.0 (new unused)—6 replicates
4. Royal Guard^®^ Benin (new unused)—6 replicates
5. Interceptor^®^ G2 (new unused)—6 replicates
6. Interceptor^®^ field net (12/24/36-months)—54 replicates
7. PermaNet^®^ 3.0 field net (12/24/36-months)—54 replicates
8. Royal Guard^®^ field net (12/24/36-months)—54 replicates
9. Interceptor^®^ G2 field net (12/24/36-months)—54 replicates

Each hut trial will last 9 weeks and will involve 9 consenting human volunteers sleeping in huts from dusk to dawn 6 days of each week of the trial. Treatments will be rotated each week using a randomized Latin Square Design to control for bias due to hut position and to minimize carry over effects between treatments. Sleepers will be rotated daily to control sleeper attractiveness to mosquitoes. On the 7th day of each week, huts will be cleaned and aired in preparation for the next rotation cycle. The trials will run for a total of 54 nights and replicate nets collected from the field will be rotated between huts on successive nights (one net per night). Nets will be made available for laboratory bioassays and chemical analysis as described earlier, immediately after they have been tested in experimental huts.

The efficacy of each ITN hut treatment at each timepoint will be measured in terms of the following outcome measures:

- Percentage reduction in mosquito entry relative to the control (deterrence)
- Percentage of mosquitoes dead after 72 h,
- Percentage of mosquitoes exiting into the veranda trap
- Percentage reduction in mosquito blood-feeding relative to the control untreated nets
- Percentage reduction in proportion of fertile surviving blood-fed mosquitoes after dissection relative to surviving blood-fed mosquitoes in the untreated control hut

WHO susceptibility bioassays will be performed to assess the susceptibility of the wild vector population at the Cove experimental hut station to the insecticides in the study nets during each hut trial.

### Experimental hut trials to compare 36 months old naturally aged field nets to washed nets

To investigate the capacity of ITN washing (20 times) to predict the performance of field aged ITNs, we will conduct another hut trial comparing nets of each ITN type washed 20 times to 36 months naturally aged field nets. ITN washing will follow WHO protocols for hut trials [13]. A total of 78 replicate 36 months old naturally aged field-collected nets of each ITN type will be tested over 78 nights. Six replicates of unwashed and washed nets of each ITN type will be tested, and each net will be given six 4 × 4cm holes following WHO guidelines. The hut trial will also be performed at the Côvè experimental hut station and will consist of the following 13 hut treatments.

1. Untreated net—6 replicates
2. Interceptor^®^ (new unused)—6 replicates
3. PermaNet^®^ 3.0 (new unused)—6 replicates
4. Royal Guard^®^ Benin (new unused)—6 replicates
5. Interceptor^®^ G2 (new unused)—6 replicates
6. Interceptor^®^ (washed 20 times)—6 replicates
7. PermaNet^®^ 3.0 (washed 20 times)—6 replicates
8. Royal Guard^®^ Benin (washed 20 times)—6 replicates
9. Interceptor^®^ G2 (washed 20 times)—6 replicates
10. Interceptor^®^ (36-months field net)—78 replicates
11. PermaNet^®^ 3.0 (36-months field net)—78 replicates
12. Royal Guard^®^ (36-months field net)—78 replicates
13. Interceptor^®^ G2 (36-months field net)—78 replicates

The trial will run for 13 weeks and will involve 13 consenting human volunteers sleeping in huts from dusk to dawn 6 days of each week of the trial. As with the other hut trials, treatments will be rotated each week using a randomized Latin Square Design and sleepers rotated daily and huts cleaned and aired on the 7th day of each week in preparation for the next rotation cycle. The trial will run for a total of 78 nights and the replicate 36 months old naturally aged field nets will be rotated between huts on successive nights (one net per night). Efficacy will be measured in terms of mosquito deterrence, mortality (72h), exiting, blood-feeding inhibition, reduction in fertility of blood-fed survivors as described earlier.

### Correlation of entomology outcomes to epidemiological endpoints

Data collected from the experimental hut trial will be used to parameterize a transmission dynamics models of malaria using previously described methods [25]. The entomological impacts of dual AI nets shall be defined by either (i) a meta-analysis of previously published experimental hut trial data, (ii) locally derived data with unwashed and washed ITNs, or (iii) locally derived data using unwashed and naturally aged field ITNs. The ability of the different models to capture the changes in malaria prevalence observed in the different study arms of the cRCT will be statistically characterized to determine whether projections based on local experimental hut trials or naturally-age field ITNs are more reliable.

### Sample size calculations

Sample size calculations are performed based on previous ITN durability data from Benin. For cohort 1, a sample size of 750 nets of each brand from 250 randomly selected households (assuming ~ 3 ITNs are distributed per household) in 5 study clusters was found sufficient to detect a 9.4% absolute difference (hazard ratio = 0.87) in ITN attrition rate assuming an attrition rate in the control arm of 70% over the 3 years and an intra-cluster correlation coefficient of 0.03. For cohort 2, a total of 522 ITNs will need to be withdrawn over three years and assuming a loss rate of 70% a total of 1800 ITNs will be marked per study arm at baseline. To reduce the impact of the durability study on the outcomes of the cRCT, cohort 2 nets will be obtained from households in the buffer areas of the selected clusters to exclude the core areas used for the cRCT sampling.

For each experimental hut trial and at each timepoint, data on the number of mosquitoes per night and the magnitude of the different random effects (huts, sleepers, nights and observational error) will be reviewed after the first round of 9 or 13 weeks and power calculations conducted by simulation using previously described methods [33]. Power analysis will determine whether the hut trial has a desired 80% power to detect superiority in mosquito killing of any new unused dual AI ITN over the new unused pyrethroid-only net, assuming a true underlying extra mortality from the dual AI net of 40%. A second round of hut trial would be performed if the desired level of power is not achieved.

### Data management

Household data collected during the census and ITN follow up surveys will be captured on electronic forms using smartphones installed with OpenDataKit (ODK) Collect while entomological data will be recorded on data entry forms and double entered pre-designed excel data bases. Throughout the study, electronic data will be stored encrypted while paper forms will be locked up in secured cabinets and will be available only to study investigators and data management staff by passwords and keys. All personal data will be anonymized using a unique identifier number for each participant and household to ensure confidentiality. At the end of the study, all electronic files and data entry forms will be respectively stored on the server and archive of the CREC-LSHTM GLP certified facility for 10 years.

### Statistical analysis

Data from the surveys at 6, 12, 24, and 36 months will be used to calculate attrition, functional survival and median survival time using Kaplan–Meier estimators. For functional survival, nets reported as given away, sold, or stolen will be excluded from the analysis. Negative binomial regression will be used to compare hole surface area between the different ITN types. Data on WHO cone bioassays and tunnel tests will be obtained from 30 nets of each product type sampled at each time point. A chi-squared test will be used to assess the proportion of nets of each ITN type passing the WHO criteria for alphacypermethrin, chlorfenapyr and pyriproxyfen bioefficacy based on combined cone and tunnel tests. Proportional outcomes (mortality, blood-feeding, exophily) from the experimental hut trials will be compared at each time point using mixed effects logistic regression, with huts, sleepers, net replicate and nightly observational error included as random or fixed effects [33]. Numerical outcomes (hut entry) will be compared using negative binomial regression. A separate model will be fitted for each outcome to determine whether there is (i) a significant change over time, and (ii) a significant difference between ITNs washed 20 times and 36-month-old naturally aged ITNs. Analysis will be performed on STATA or the package *lme4* of the R software.

### Ethical considerations

Ethical approval for this study has been obtained from the ethics review boards of the Ministry of Health in Benin (N°6/30/MS/DC/DRFMT/CNERS/SA), the institutional review board of the London School of Hygiene and Tropical Medicine (N°16,237), and the WHO Research Ethics Review Committee (ERC.0003153). The cRCT is registered on clinicaltrials.gov (NCT03931473).

Heads of households involved in the durability study will give informed consent prior to their participation in the study. The consent form will be written in French and explained to them in their local language. Where the individual is unable to read or write, their fingerprint will be taken, and a signature obtained from a witness to the informed consent procedure. All personal data will be anonymized prior to data processing. Written informed consent will also be obtained from all human volunteer sleepers for experimental hut trials prior to participation. Sleepers will be offered a free course of chemoprophylaxis spanning the duration of the study and 4 weeks following its completion to mitigate malaria infection risk. Approval for use of guinea pigs for tunnel tests has been obtained from the LSHTM Animal Welfare Ethics Review Board (Ref: 2020–01). Guinea pig colonies will be maintained according to institutional standard operating procedures (SOPs) developed in line with relevant national regulation and UK Research and Innovation policies for the use of animals for scientific research purposes.

## Discussion

This paper describes the methodology for evaluating the physical and insecticidal durability of two dual AI ITNs (Interceptor^®^ G2 and Royal Guard^®^) as part of an ongoing cRCT in Benin. Both nets are being compared to a WHO prequalified alpha-cypermethrin net (Interceptor^®^). By investigating the attrition and fabric integrity of the study nets, we will generate data needed for planning their replacement and for making programmatic and procurement decisions [14] once they are deployed more widely. Previous studies have shown that householders distinguish between ITN products and tend to overwhelmingly prefer polyester ITNs over polyethylene ITNs [34]. By investigating householders’ preferences and perceptions of the different ITN fabric types used in the formulation of the study nets (polyethylene for Royal Guard^®^ and polyester for Interceptor^®^ G2 and Interceptor^®^), we will also provide more insights into how fabric type may affect ITN retention and overall impact in Benin.

Dual AI ITNs cost significantly more than standard pyrethroid nets due to the additional non-pyrethroid active ingredient on these nets. To ensure value for money, it is essential to closely monitor the insecticidal activity of the non-pyrethroid component in these nets using insecticide durability bioassays. Current WHO ITN insecticide durability guidelines were developed mostly for pyrethroid-only nets hence the methods and recommended strains were suited to their rapid mode of action [13]. We propose subtle modifications to these standard laboratory bioassay methods and to recently developed consensus SOPs [35] to facilitate monitoring of the insecticidal durability of chlorfenapyr in Interceptor^®^ G2 and pyriproxyfen in Royal Guard® while improving the efficiency of the bioassays. First, the mosquito strains used in laboratory bioassays with dual AI ITNs must allow assessment of the non-pyrethroid insecticide in the net without interference from the pyrethroid component. For assessment of pyriproxyfen in Royal Guard® for example, it is essential that enough blood-fed survivors are obtained post ITN exposure. By using a high intensity pyrethroid-resistant strain (*An. coluzzii* Akron) we will substantially reduce the toxicity and confounding effect of the pyrethroid allowing a more realistic assessment of the non-pyrethroid components.

For Interceptor^®^ G2 bioassays, we will perform only tunnel tests with the pyrethroid resistant Akron strain focusing primarily on evaluating the activity of the chlorfenapyr component of the net. Chlorfenapyr acts on the respiratory system of mosquito vectors hence its activity against malaria mosquitoes is optimal when they are active and host-seeking at night in line with their nocturnal behaviour [19]. The tunnel test being an overnight bioassay that allows free mosquito movement therefore constitutes a more appropriate test method for evaluating the efficacy of chlorfenapyr in Interceptor^®^ G2 compared to daytime 3 min cone bioassays. In addition, because the alpha-cypermethrin component in Interceptor^®^ G2 is relatively low (~ 50% of what is typically applied on ITNs) its insecticidal activity would likely be more limited and less durable even in cone bioassays. The chlorfenapyr component is therefore expected to maintain the killing effect of these nets as they age. By testing Interceptor^®^ G2 nets only in tunnels we will ensure high efficiency of the laboratory bioassays.

For Royal Guard^®^, the insecticidal durability of the alpha-cypermethrin component will be investigated following current WHO ITN guidelines while modified bioassay methods will be used to assess the activity of pyriproxyfen. Pyriproxyfen acts by sterilizing adult mosquitoes and based on this mode of action, two different methods have been proposed for investigating its impact on ITNs; assessment of oviposition inhibition and dissection of mosquito ovaries to determine their developmental stage [20, 22, 35, 36]. The oviposition inhibition method directly measures the impact of pyriproxyfen on mosquito offspring by holding exposed blood-fed mosquitoes in oviposition chambers to give them opportunity to lay eggs and determining the proportional reduction in numbers laying relative to unexposed mosquitoes. The dissection method is based on the physiological impact of pyriproxyfen on the ovarian development of adult female mosquitoes leaving them visibly damaged and halting their follicular maturation process [37]. With this method, mosquito ovaries are dissected, and their developmental stage assessed to determine whether they are fertile or not; mosquitoes with stage V fully developed ovaries are considered fertile. While both methods have been effectively used to measure the impact of pyriproxyfen on nets [20, 36], preliminary studies comparing them demonstrated that the dissection method was more reproducible, more sensitive and less time consuming (unpublished data). Hence, given the volume of bioassays envisaged in this study, the mosquito ovary dissection method will provide a higher throughput alternative for assessing the sterilizing effect of pyriproxyfen in Royal Guard®. In addition to its sterilizing effects, pyriproxyfen has also been demonstrated to shorten the lifespan of adult mosquitoes when applied on ITNs [38]. However, the evaluation of this life shortening effect in laboratory bioassays requires that mosquitoes are observed for several weeks post-exposure which would be very time consuming and too demanding to be performed in routine ITN insecticidal durability studies.

Current WHO ITN evaluation guidelines define specific thresholds for determining the efficacy of ITNs in insecticidal durability bioassays. Nets that induce ≥ 95% knockdown and/or ≥ 80% mortality in cone bioassays or ≥ 80% mortality and/or ≥ 90% blood feeding inhibition in tunnel tests are considered efficacious. While proposing these same efficacy criteria for the laboratory bioassays for alpha-cypermethrin and chlorfenapyr bioefficacy in the study nets, it is recognized that these cut-offs were primarily developed for fast-acting pyrethroid insecticides on nets and may need to be modified for non-pyrethroid insecticides. Indeed, new draft WHO guidelines proposed for ITN prequalification aim more for a weight of evidence approach and do not allude to specific efficacy thresholds [39]. The weight of evidence approach may be suitable for assessing individual ITN products but could be challenging for comparing between ITN product brands and making programmatic and procurement decisions, which is usually the main objective for ITN durability studies. Efficacy criteria for the sterilizing effects of pyriproxyfen on ITNs are yet to be defined in WHO guidelines. Further studies investigating correlations between the sterilizing effects of Royal Guard^®^ nets in laboratory bioassays and their epidemiological performance against clinical malaria in the current trial, may contribute towards the identification of a suitable cut-off for the impact of pyriproxyfen in ITN durability studies.

Despite the methodological modifications we propose for laboratory bioassays in this study, it is unclear whether typical ITN laboratory bioassay techniques (cone bioassays and tunnel tests) would be sufficiently informative of the insecticidal durability of dual AI ITNs as these methods were developed for pyrethroid-only ITNs [22, 23]. Other bioassay methods under consideration for dual AI ITNs are the WHO wire ball assay [13, 40] and the net-in-tube test [22] that provide a more forced contact between the mosquito and the net potentially resulting in greater levels of AI pick up from the net than in cone bioassays. Given the mode of action of chlorfenapyr described earlier, these assays would be less suitable for the assessment of its activity in Interceptor^®^ G2 as they provide less opportunity for mosquito movement and host seeking. In addition, our preliminary studies assessing the baseline activity of pyriproxyfen in Royal Guard^®^ using the net-in-tube method resulted in higher levels of mosquito mortality with high intensity pyrethroid-resistant strains leaving very few blood-fed survivors for ovarian dissection. These methods due to the forced contact they provide may therefore be less suitable for monitoring changes in the bioefficacy of the pyriproxyfen component in Royal Guard^®^ over time. Experimental hut trials are more representative of real-life settings and are widely used for evaluating ITNs under semi-field conditions for product development and for decision-making about their deployment [41]. In these trials, ITNs are tested overnight against wild free-flying vector mosquitoes entering naturally into the huts in the presence of a human volunteer and the levels of blood-feeding inhibition and mortality rates achieved are used as proxies for their capacity to provide personal and community protection to users. Hence the assessment of field collected ITNs in experimental hut trials proposed in this study will provide additional insights into the impact of these dual AI ITNs against malaria as they age under operational conditions.

In standard ITN experimental hut evaluations, the performance of nets washed 20 times is widely accepted as a measure of their efficacy after 3 years of field use and ITN products have been prequalified by WHO based on their performance after 20 washes. However, not much is known about how this washing process and number of washes relates to the natural ageing of nets in households. The hut trial comparing naturally aged nets to washed nets in our study provides an opportunity to investigate this claim. To further support this objective, the number of times householders’ wash their nets over 3 years of household use and the washing methods used will also be investigated. Another key objective of this study is to use mathematical models to determine whether the entomological performance of ITNs in the experimental hut trials performed in this study could be predictive of their performance in the cRCT. The findings from this modelling study will provide further insights into the role of experimental hut trials in the dual ITN product development process.

## Conclusion

This study will provide information on the durability of two dual AI nets (Interceptor^®^ G2 and Royal Guard^®^) in Benin and will help identify suitable methods that could contribute towards standardized guidelines for monitoring their insecticidal activity under operational conditions. The modelling component will help determine the capacity of experimental hut trials to predict the epidemiological performance of dual AI nets.

## Status of study

Ethical approval has been obtained and ITNs distributed in 2020. ITN follow up surveys have been completed up to 24 months post-ITN distribution. Laboratory bioassays and hut trials with nets withdrawn up to 24 months post-ITN distribution are on-going. The complete data sets from the study should be available in 2024.

## Data Availability

Not applicable.

## Abbreviations

PBO: Piperonyl butoxide
ITN: Insecticide-treated nets
LLIN: Long-lasting insecticidal nets
WHO: World Health Organization
PQ: Prequalification team
AI: Active ingredient
GLP: Good Laboratory Practice
cRCT: Cluster randomized controlled trial
CREC: Centre de Recherche Entomologique de Cotonou
LSHTM: London School of Hygiene & Tropical Medicine
PAMVERC: Pan African Malaria Vector Research Consortium
